# The prognostic value of the serum neuron specific enolase and lactate dehydrogenase in small cell lung cancer patients receiving first-line platinum-based chemotherapy

**DOI:** 10.1097/MD.0000000000008258

**Published:** 2017-11-17

**Authors:** Xiaofan Liu, Weiming Zhang, Wen Yin, Yang Xiao, Changzhi Zhou, Yi Hu, Shuang Geng

**Affiliations:** Department of Respiratory, The Central Hospital of Wuhan, Tongji Medical College, Huazhong University of Science and Technology, Wuhan, Hubei, China.

**Keywords:** first line, lactate dehydrogenase (LDH), platinum-based chemotherapy, prognostic, serum neuron specific enolase (NSE), small cell lung cancer (SCLC)

## Abstract

The aim of this study was to investigate the associations of serum levels of neuron-specific enolase (NSE), pro-gastrin releasing peptide (ProGRP), and lactate dehydrogenase (LDH) with clinical response and survival in small cell lung cancer (SCLC) patients receiving first-line platinum-based chemotherapy.

One hundred thirty-six patients with SCLC were recruited in this study. All the patients received first-line platinum-based chemotherapy. Clinical efficacy was assessed according to Response Evaluation Criteria in Solid Tumors v1.1 criteria. Serum samples were collected from SCLC patients before chemotherapy. NSE, ProGRP, and LDH levels were measured by commercial electrochemiluminescence immunoassay, enzyme-linked immune sorbent assay, and kinetic spectrophotometric method, respectively.

Overall response rate was 71.3% with 97 patients who achieved complete response (CR) +  partial response (PR). NSE and LDH level declined in patients who achieved CR + PR compared with patients in stable disease (SD) and progress disease (PD). Multivariate logistic regression analysis revealed that NSE > 50.324 ng/mL, stage ED, and distant metastases were independent risk factors for patients achieving CR + PR, and chemotherapy > 4 cycles was an independent protective factor in predicting CR + PR. Receiver operating characteristic (ROC) curves presented that expression of NSE, ProGRP, and LDH are of good predicting value for patients achieving CR + PR. Patients with a higher level of NSE and LDH presented worse progression-free survival and overall survival. In addition, multivariate Cox regression analysis showed that NSE level > 50.324 ng/mL and distant metastasis were independently correlated with worse OS.

Serum NSE and LDH could be promising biomarkers for predicting therapy response and survival of SCLC patients receiving first-line platinum-based chemotherapy.

## 1 Introduction

Lung cancer, the leading cause of cancer deaths worldwide, results in 1.6 million deaths every year.^[1–4]^ Small cell lung cancer (SCLC), accounts for approximately 13% to 20% of all lung cancer cases, and is characterized by its rapid disease progression and propensity for early metastases.^[3,5]^ About two-third SCLC patients present with metastasis in regional lymph nodes or distant organs at the time of diagnosis, which dramatically declines remission rate of this extremely aggressive malignancy.^[6,7]^ Despite SCLC is highly sensitive to chemotherapy and radiotherapy, with initial response rates from 60% to 80%,^[8]^ relapses still exist in patients.^[3,5]^ Median survival (MS) for SCLC patients with limited disease (LD) is currently 14 to 20 months, with 20% to 40% surviving to 2 years, and for those with extensive disease (ED), the values are 7 to 13 months and 5%, respectively.^[3,5,9]^

Combination chemotherapy, generally 4 to 6 cycles of platinum-based and etoposide or irinotecan, is the cornerstone treatment in both LD and ED SCLC patients, especially for patients with metastatic disease.^[3,8]^ A phase III randomized trial revealed that patients receiving platinum-based chemotherapy had better clinical response and longer survival duration than patients treated with anthracycline-based chemotherapy.^[10]^ Regardless of these improvements in SLCL treatments, 19% to 43% patients still do not response well to platinum-based chemotherapy,^[10,11]^ thus identification of convincing biomarkers for prognosis is deadly needed.

With neuroendocrine cellular origin, SCLC usually expresses neuroendocrine markers, including neuron-specific enolase (NSE), chromogranin A, pro-gastrin releasing peptide (ProGRP), bombesin, and lactate dehydrogenase (LDH).^[5]^ NSE, known as enolase-γ, is a dimer composed of apparently identical subunits, with high concentrations localized in neurons and neuroendocrine cells.^[12]^ Accumulating researches demonstrate that NSE plays critical roles in aerobic glycolysis of various tumors such as SCLC.^[6,13]^ Apart from the correlation of high NSE levels and tumor mass extension, NSE levels are also reported to be frequently reduced in SCLC after chemotherapy treatment.^[5]^ ProGRP, a gastrointestinal hormone of the bombesin family, is the precursor for gastrin-releasing peptide (GRP) and more stable than GRP.^[5,14]^ ProGRP discloses high sensitivity and specificity in diagnosing SCLC and is associated with disease stage as well as distant metastasis.^[5]^ As for LDH, it is a protein related to tumor metabolism and can be applied for the detection of malignancies in serum. Specifically, LDH is the key enzyme in glycolytic metabolism that stimulates the interconversion of pyruvate to lactate.^[6]^ In addition, several investigators found that LDH levels are elevated during cell malignant transformation in various types of cancer, including SCLC.^[15,16]^ However, few reports illuminate the prognostic value of NSE, ProGRP, and LDH in SCLC patients applying first-line platinum-based chemotherapy.

Our study aimed to investigate the association of serum levels of NSE, ProGRP, and LDH with clinical response and survival in SCLC patients receiving first-line platinum-based chemotherapy.

## 2 Methods

### 2.1 Participants

One hundred thirty-six patients with SCLC were recruited from September 1, 2012, to December 31, 2014, at the Department of Respiratory in The Central Hospital of Wuhan, Tongji Medical College, Huazhong University of Science and Technology. All the patients were diagnosed by clinical, pathological, and radiographic evidences, and no treatments were performed before joining this study. Patients less than 18, with tumor or severe infection history, lack compliance, pregnancy, or lactation were excluded. Staging was carried out according to the veterans administration lung cancer group (VALG) staging system: LD was defined as disease confined to 1 hemithorax including mediastinal lymph nodes and/or supraclavicular lymph nodes; ED was defined in those not having LD and in patients with a malignant pleural effusion.^[17]^ This study was approved by Ethics Committee of The Central Hospital of Wuhan, Tongji Medical College, Huazhong University of Science and Technology, and all the participants (SLCL patients and HCs) signed the informed consents.

### 2.2 Treatments and follow-ups

All the patients received first-line platinum-based chemotherapy. Patients in LD also received radiotherapy. And patients were followed up once a month in first half year, and once every 3 months from 0.5 year to 2 years, then once every 6 months from 2 years to the last date of follow-up (December 31, 2016). The median follow-up time was 29 months.

### 2.3 Measurements

Clinical efficacy was assessed according to Response Evaluation Criteria in Solid Tumors (RECIST) v1.1 criteria as follows: CR (complete response), PR (partial response), PD (progress disease), and SD (stable disease). Overall response rate (ORR) was defined as the percentage of patients who achieved CR + PR. And progression-free survival (PFS) was calculated from the chemotherapy to time of first progression or death from any cause. Overall survival (OS) was also calculated from the chemotherapy to time of death from any cause.

### 2.4 Samples and measurement of NSE, ProGRP, and LDH

Serum samples were collected from SCLC patients before chemotherapy. NSE was measured by commercial electrochemiluminescence immunoassay (ECLIA) using ELECSYS 2010 analyzer and reagent kits (Roche Diagnostics, Mannheim, Germany). ProGRP was determined by enzyme-linked immune sorbent assay (ELISA) using ELISA kit (CUSABIO CORP, Wuhan, China). The LDH activity detected by a kinetic spectrophotometric method using Model 912 biochemical analyzer (Roche Diagnostics, Mannheim, Germany).

### 2.5 Statistics

SPSS 19.0 (IBM Corp., Ltd, New York) was used for statistical analysis in this study. Data were presented as mean with standard deviation, or median with one-fourth quarter to three-fourth quarter, or count with percentage. The Kolmogorov–Smirnov test was used to assess the normality of continuous data. Difference among 3 groups was determined by Kruskal–Wallis H rank sum test. Receiver operating characteristic (ROC) curve was performed to evaluate serum NSE, ProGRP, and LDH levels for predicting CR + PR by first-line chemotherapy, and univariate and multivariate logistic regression was used to analyze the factors for predicting CR + PR. In addition, Kaplan–Meier (K-M) curve and Log-Rank test was used to investigate the correlation of serum NSE, ProGRP, and LDH levels with PFS as well as OS, and univariate and multivariate logistic regression was carried out to analyze the factors that influence PFS and OS. The correlations among the levels of NSE, ProGRP, and LDH before chemotherapy were determined by Spearman test. *P* value < .05 was considered significant.

## 3 Results

### 3.1 Characteristics

**Figure 1:**
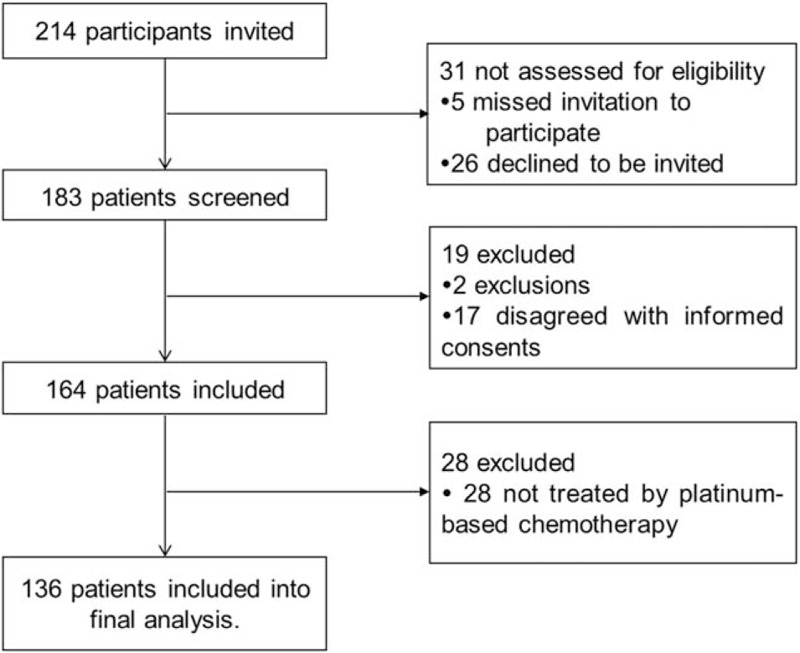
Study flow.

During study period, 214 SCLC patients were invited at the beginning and 31 cases were not assessed for eligibility, among which 5 patients missed invitation and 26 patients declined to be invited (Fig. 1**)**. In the remaining 183 patients who were screened for eligibility, 19 cases were excluded: 2 patients fit for the exclusion criteria and 17 disagreed with informed consents, leading to 164 patients included. Ultimately, of the 164 included patients, 28 cases did not receive first-line platinum-based chemotherapy, resulting in 136 patients included in final analysis.

Mean age of the total 136 SCLC patients was 53.3 ± 10.7 years (Table 1). And there were 88 (64.7%) males and 48 (35.3%) females. Among all SCLC patients, 42 (30.9%) patients had smoking history. As to disease stage, 77 (56.6%) patients were in LD stage, while 59 (43.4%) patients were in ED stage. Seventy-six (55.9%) patients were treated with >4 cycles of platinum-based chemotherapy, while 60 (44%) patients were treated with ≤ 4 cycles of platinum-based chemotherapy. In addition, 91 (66.9%) patients were with larger tumor size (>3 cm), and 45 (33.1%) patients were with smaller tumor size (≤3 cm), while the number of patients with metastasis were 48 (35.3%).

**Table 1:**
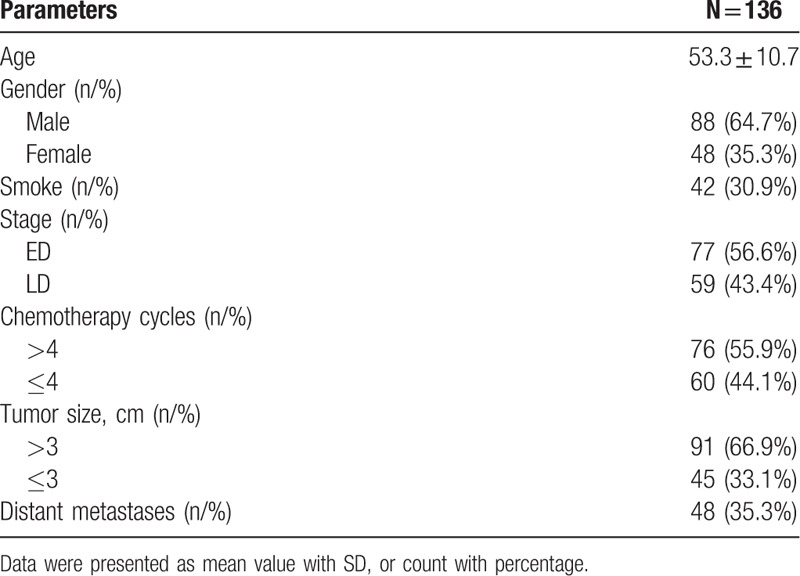
Characteristics of SCLC patients.

### 3.2 Clinical efficacy

Post first-line platinum-based chemotherapy, 2 (1.5%) patients achieved CR and 95 (69.9%) patients achieved PR as listed in Table 2. Above all, ORR was 71.3% with 97 patients who achieved CR + PR, in which 51 (86.4%) patients were LD stage and 46 (59.7%) patients were ED stage. While for the remaining patients, there were 32 (23.5%) patients with SD and 7 (5.1%) patients with PD. Other data of clinical efficacy are summarized in Table 2.

**Table 2:**
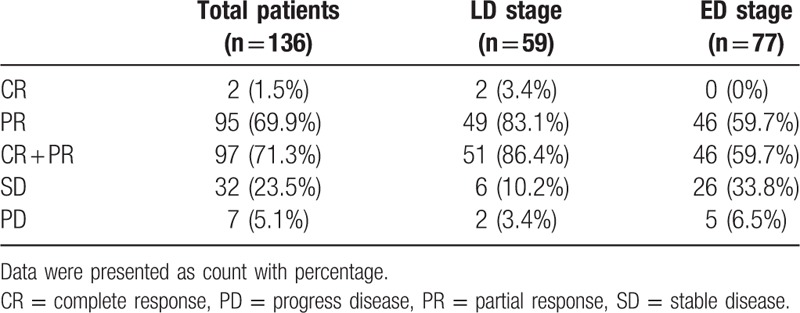
Clinical efficacy by first-line chemotherapy in SCLC patients.

### 3.3 Correlation of NSE, ProGRP, and LDH levels with clinical efficacy

Serum level of NSE declined in patients achieved CR + PR [40.495 (21.597–70.224) ng/mL] compared with patients were SD and PD [69.253 (36.800–109.700) ng/mL and 84.211 (65.726–135.623) ng/mL, respectively] (Table 3). The LDH levels before chemotherapy in patients who achieved CR + PR were markedly lower than that in patients were SD and PD [179.90 (97.56–301.36) IU/L vs 244.22 (130.44–424.51) IU/L and 339.77 (223.51–528.40) IU/L]. However, no difference of ProGRP level in patients in different therapy responses was found.

**Table 3:**
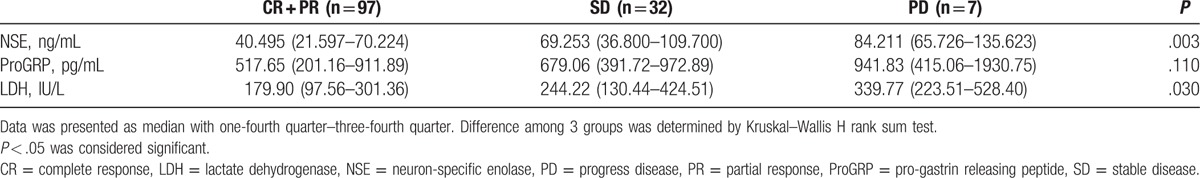
Correlation of NSE, ProGRP, and LDH levels with clinical efficacy.

### 3.4 Analysis of factors affecting clinical efficacy

As summarized in Table 4, NSE and ProGRP levels were divided into 2 groups by the median value as cut-off point, respectively, while LDH levels were also divided into 2 groups by the value of 200 IU/L (<200 IU/L was defined as normal, while ≥200 IU/L was abnormal). Univariate logistic regression analysis showed that patients with a higher level of NSE (*P* = .002), stage ED (*P* = .001), and distant metastases (*P* = .040) were less likely to achieve CR + PR, while chemotherapy > 4 cycles (*P* = .029) was a promoting factor for patients achieving CR + PR. All factors with *P* value < .1 were subsequently included in multivariate logistic regression analysis, which revealed that a high level of NSE (*P* = .004), stage ED (*P* = .003), and distant metastases (*P* = .017) could independently predict that patients were less likely to achieve CR + PR, and chemotherapy > 4 cycles (*P* = .040) was an independent factor in predicting patients achieving CR + PR.

**Table 4:**
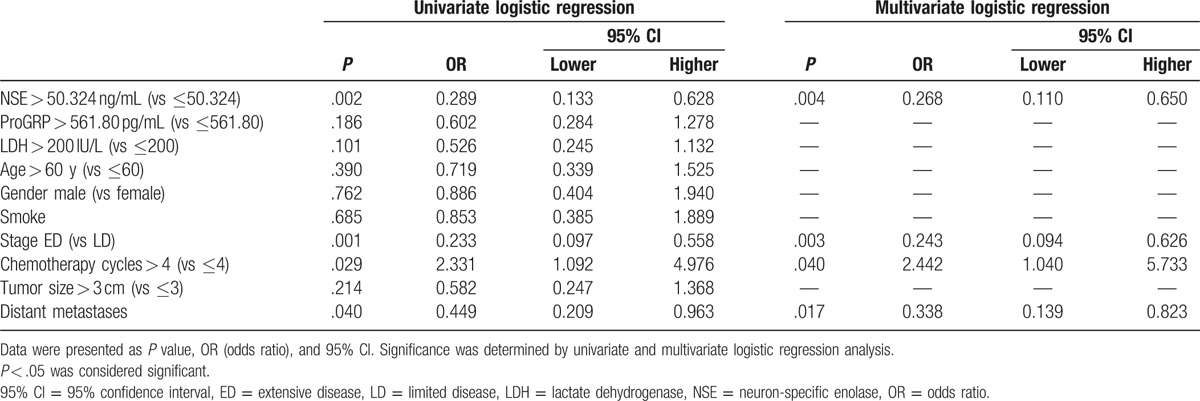
Analysis of factors influenced CR + PR.

**Figure 2:**
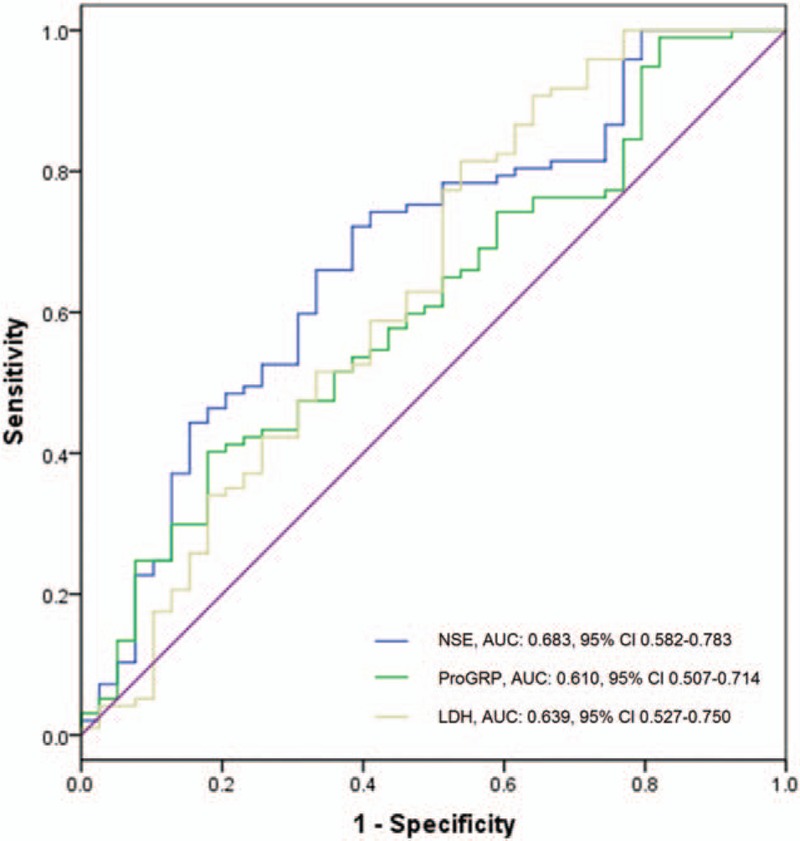
ROC curves of NSE, ProGRP, and LDH for SCLC. The analysis was determined by ROC curve analysis.

ROC curves presented that expression of NSE, ProGRP, and LDH was of good predicting value for patients achieving CR + PR with the area under curve (AUC) of 0.683 [95% confidence interval (95% CI): 0.583–0.783], 0.610 (95% CI: 0.507–0.714), and 0.639 (95% CI: 0.527–750), respectively (Fig. 2).

### 3.5 The association of NSE, ProGRP, and LDH levels with PFS and OS

**Figure 3:**
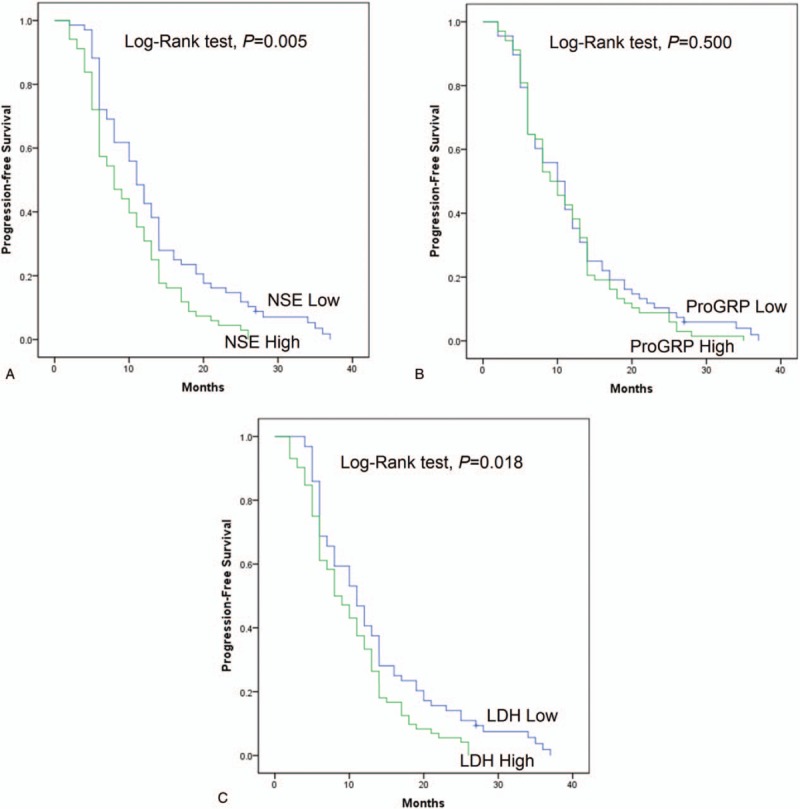
PFS of SCLC patients receiving first-line platinum-based chemotherapy with high or low levels of NSE, ProGRP, and LDH serum levels. (A) PFS of SCLC patients receiving first-line platinum-based chemotherapy with high or low levels of NSE. (B) PFS of SCLC patients receiving first-line platinum-based chemotherapy with high or low levels of ProGRP. (C) PFS of SCLC patients receiving first-line platinum-based chemotherapy with high or low levels of LDH. PFS in different groups were analyzed by Kaplan–Meier curve and log-rank test. *P* < .05 was considered significant.

To detect the association of NSE, ProGRP, and LDH levels with PFS and OS, we proceeded with K-M curves. As shown in Fig. 3A, patients with higher serum level of NSE exhibited worse PFS compared with patients with lower serum level of NSE. Similarly, patients with higher expression of LDH in serum also presented shorter PFS (Fig. 3C). However, no difference of PFS for patients was found in high and low ProGRP level groups (Fig. 3B).

**Figure 4:**
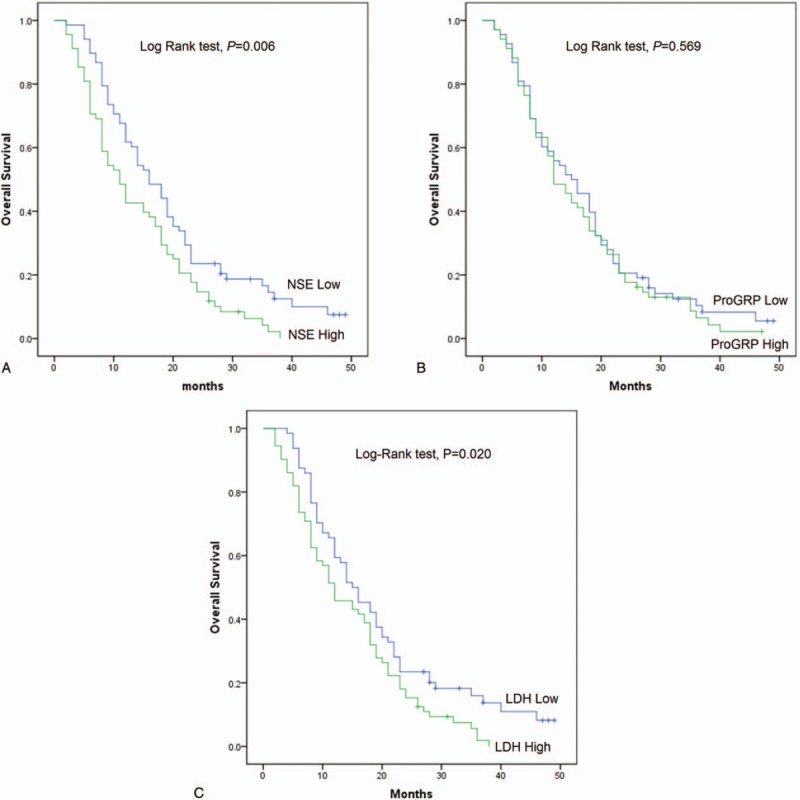
OS of SCLC patients receiving first-line platinum-based chemotherapy with high or low levels of NSE, ProGRP, and LDH serum levels. (A) OS of SCLC patients receiving first-line platinum-based chemotherapy with high or low levels of NSE. (B) OS of SCLC patients receiving first-line platinum-based chemotherapy with high or low levels of ProGRP. (C) OS of SCLC patients receiving first-line platinum-based chemotherapy with high or low levels of LDH. OS in different groups were analyzed by Kaplan–Meier curve and log-rank test. *P* < .05 was considered significant.

As presented in Fig. 4, similar associations of NSE, ProGRP, and LDH serum levels with OS were discovered. Patients with higher serum levels of NSE and LDH were with poorer OS (Fig. 4A, C), while no correlation of ProGRP expression in serum with PFS of patients was found (Fig. 4B).

### 3.6 Analysis of factors affecting PFS and OS

To investigate factors influencing PFS, univariate Cox hazard regression was conducted. And high expression of NSE (*P* = .008), high level of LDH > 200 IU/L (*P* = .027), stage ED (*P* = .008), distant metastasis (*P* = .025), and chemotherapy cycles≤4 (*P* = .034) were factors in predicting worse PFS, while ProGRP (*P* = .526), age (*P* = .526), gender (*P* = .649), smoking history (*P* = .151), and tumor size (*P* = .240) were not correlated with PFS (Table 5). Afterwards, all factors with *P* value < .1 were analyzed by multivariate Cox hazard regression analysis, and NSE level > 50.324 ng/mL (*P* = .016), LDH > 200 IU/L (*P* = .010), and distant metastasis (*P* = .017) were manifested to be independently correlated with shorter PFS (Table 5).

**Table 5:**
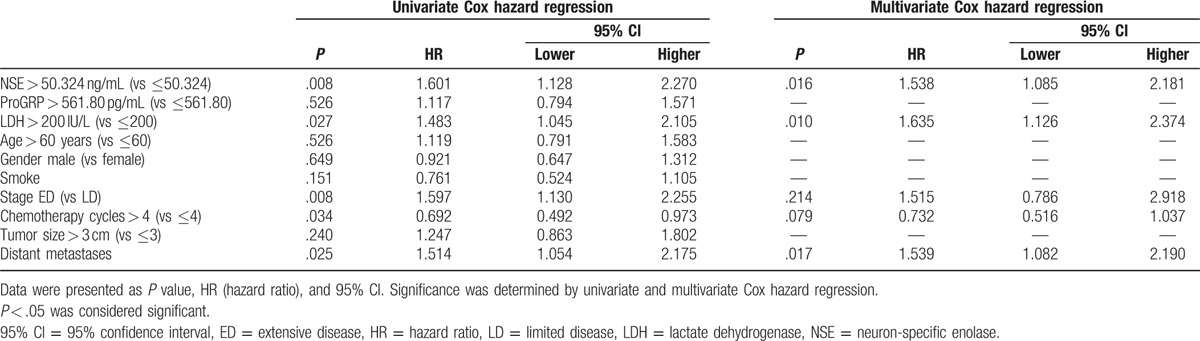
Analysis of factors influenced PFS.

As presented in Table 6, univariate Cox hazard regression was performed to analyze the predicting factors for risk of shorter OS. Analysis showed that NSE level > 50.324 ng/mL (*P* = .009) and LDH > 200 IU/L (*P* = .025), stage ED (*P* = .010) as well as distant metastasis (*P* = .017) were prognostic factors for shorter OS. Subsequently, factors with *P* value < .1 were analyzed by multivariate Cox hazard regression, which revealed that NSE level > 50.324 ng/mL (*P* = .019) and distant metastasis (*P* = .008) were independently correlated with worse OS (Table 6).

**Table 6:**
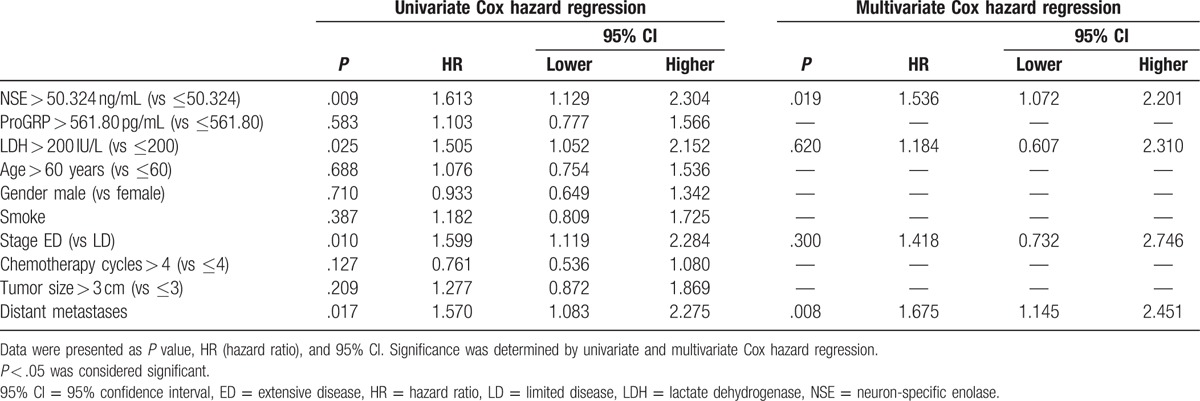
Analysis of factors influenced OS.

### 3.7 Correlations among NSE, proGRP, and LDH levels before chemotherapy

**Figure 5:**
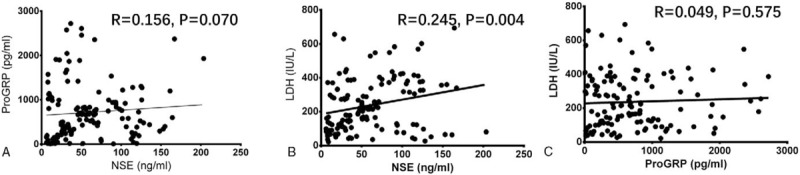
Correlations among NSE, ProGRP, and LDH levels before chemotherapy. (A) Correlation of NSE level with ProGRP level before chemotherapy. (B) Correlation of NSE level with LDH level before chemotherapy. (C) Correlation of ProGRP level with LDH level before chemotherapy. Comparison between 2 groups was determined by Spearman test. *P* < .05 was considered significant.

The NSE expression before chemotherapy was positively associated with the level of LDH pre-chemotherapy (*R* = .245, *P* = .004) (Fig. 5B). While as presented in Fig. 5A and C, no correlation of proGRP expression with NSE (*R* = 0.156, *P* = .070) and LDH (*R* = 0.049, *P* = .575) was found.

## 4 Discussion

In this study, after first-line platinum-based chemotherapy, ORR was 71.3% with 97 patients who achieved CR + PR; decreased levels of NSE and LDH were observed in patients who achieved CR + PR, and a high level of NSE, stage ED, and distant metastases could independently predict patients who were less likely to achieve CR + PR, and chemotherapy > 4 cycles was an independent factor in predicting patients achieving CR + PR; patients with overexpression of serum NSE and LDH exhibited shorter PFS and OS; in addition, high expression of NSE, high level of LDH, and distant metastasis were manifested to be independently correlated with shorter PFS, while a high level of NSE and distant metastasis were independent predicting factors for worse OS.

SCLC is an aggressive cancer, with high incidence, mortality, and unfavorable prognosis.^[2,3]^ The first symptoms of SCLC, such as cough, wheeze, and dyspnea, were not long in making its appearance, with the duration before presentation currently being 8 to 12 weeks.^[3]^ Improved survival remains the ultimate goal; however, new drug combinations and approaches only have made little difference to PFS, as well as OS, especially for patients in ED stage.^[3]^ The choice of first-line treatment for SCLC patients in ED stage remains 4 to 6 cycles of platinum-based chemotherapy, such as etoposide combined with a platinum salt (cisplatin or carboplatin).^[3]^ Therefore, prognostic tools to monitor the treatment and to early assess the therapy response during first-line chemotherapy are eagerly needed to be identified, which would help to tailor and optimize disease management for SCLC patients.

Tumor markers were known as a class of biologically active substances, which were synthesized by tumor cells and then released into the body fluids or tissues.^[5]^ Recently, identification of the optimal tumor makers for SCLC prognosis has been the focus of many researches. Previous studies have already presented that NSE has a potential role of mirroring the SCLC disease activity and metabolism, and then was applied to early predict insufficient response to chemotherapy.^[5,6,14,15,18]^ As one of the most powerful prognostic markers for SCLC, NSE has been widely and preferentially used in clinical trial and routine practice.^[5,18,19]^

NSE was firstly discovered in brain tissues and in amine precursor uptake and decarboxylation (AUPD) cells subsequently, while a former study has illuminated that serum NSE can be produced by SCLC tumor as well.^[20,21]^ The role of NSE level in assessing the therapy response has also been characterized by other studies, a study conducted on SCLC patients elucidated a prognostic value of NSE for relapse, before which patients had achieved CR or PR to first-line chemotherapy.^[22,23]^ For the prognostic value of NSE, Ebert et al^[24]^ discovered that patients with a decrease of NSE level had obviously better survival than those with relatively higher levels of NSE. As another tumor marker, serum LDH is elevated during cell malignant transformation.^[15,25,26]^ In this present study, the results showed that high serum LDH level was correlated with unsatisfactory therapy response and poorer survival of SCLC patients, indicating that LDH is a potential marker in prognosis of SCLC in predicting the efficacy of chemotherapy and survival for SCLC patients. Numerical studies indicated that LDH expression increased with tumor progression, and the prognostic role of LDH in cancers has been reported in respectable studies.^[15,25]^ Hermes et al^[27]^ identified LDH level as an independent predictor of mortality and morbidity fin SCLC patients under chemotherapy. LDH expression in serum was also demonstrated to be a predicting factor for therapy response and survival of SCLC patients treated with chemotherapy in other studies.^[28–30]^ Our study revealed that lower serum levels of NSE and LDH in SCLC patients were associated with better therapy response and survival; those data were broadly in line with this study. In addition, high level of NSE, stage ED, and distant metastases could independently predict worse CR + PR in patients according to multivariate logistic regression analysis. Although the measurements of stages and distant metastases are relatively established method for predicting outcome of patients, there is still an additional benefit of NSE measurement over stage and distant metastases. Stages and distant metastases measurements are not applicable for all SCLC patients because the measurements are invasive, while the examination of NSE in serum is much more easy to perform on patients in clinical practice, which is conducted through collecting blood samples, when compared with the measurements of stage and distant metastases.

ProGRP is known as a precursor of GRP and was proved to be more stable than GRP; former studies validated that alteration of ProGRP level could be a prognostic factor for clinical response, PFS, and OS of SCLC patients.^[31]^ However, the role of ProGRP in distinguishing good and poor prognosis was not revealed in our study. And many factors may be the potential roots of discrepancy, such as different treatments and clinical characteristics of patients, the varying techniques used to detect ProGRP levels, and the threshold levels of ProGRP. In addition, some researches have also previously illustrated that ProGRP had a worse sensitivity than NSE in terms of prognostic value for SCLC patients.^[5,32]^

Some limitations existed in our study: in this prospective single-armed study, the sample size that included 136 SCLC patients is relatively small, which means a large sample size study is needed in the future; the cut-off value of NSE and ProGRP in our study was the median value, which might result in some bias in this study; and the median follow-up was 29 months, which is a relatively short follow-up period. However, survival of SCLC patients is poor, which resulted in the difficulty in follow-up.

## 5 Conclusion

Our study validated that both serum NSE and LDH could be regarded as promising biomarkers for predicting therapy response and survival of SCLC patients receiving first-line platinum-based chemotherapy.
